# Association of Baseline Depressive Tendency with Post-Counseling Clinical Status in Patients with Thymoma-Associated Myasthenia Gravis

**DOI:** 10.3390/healthcare14162577

**Published:** 2026-08-17

**Authors:** Ching-Fu Yeh, Wen-Han Chang, Chen-Yun Cheng, Jiann-Horng Yeh, Ming-Hsing Chang

**Affiliations:** 1 Department of Neurology, Shin Kong Wu Ho-Su Memorial Hospital, Taipei 111045, Taiwan; cfyeh0326@gmail.com (C.-F.Y.); m001074@ms.skh.org.tw (J.-H.Y.); 2 Taipei Branch Office, Teacher Chang Foundation, Taipei 104036, Taiwan; wenhang64@gmail.com; 3 Institute of Public Health, School of Medicine, National Yang Ming Chiao Tung University (NYCU), Taipei 112304, Taiwan; cheng.md10@nycu.edu.tw; 4 School of Medicine, College of Medicine, Fu Jen Catholic University, New Taipei City 242062, Taiwan

**Keywords:** myasthenia gravis, thymoma, depressive tendency, psychological well-being, mental health BMI

## Abstract

**Background:** Depressive symptoms are highly prevalent among patients with thymoma-associated myasthenia gravis (MG) and are associated with poorer clinical status and quality of life. However, their relationship with psychological counseling outcomes remains insufficiently investigated. **Objectives**: This study aimed to examine the association between baseline depressive tendency, baseline clinical status, and post-counseling clinical outcomes in patients with thymoma-associated MG. **Methods**: This single-group pre-post exploratory pilot study enrolled 17 patients with thymoma-associated MG who received eight sessions of Gestalt-oriented psychological counseling. A depressive tendency was defined as a baseline Patient Health Questionnaire-9 (PHQ-9) score ≥10 (*n* = 6, 35.3%). Clinical outcomes, including the Myasthenia Gravis Activities of Daily Living scale (MG-ADL), Myasthenia Gravis Quality of Life 15-item scale (MG-QoL15), mental BMI (mBMI), and PHQ-9 scores, were assessed before and after the intervention. Multivariable linear regression analyses were performed using baseline depressive tendency as the primary predictor of each post-counseling outcome. The models were sequentially adjusted for the corresponding baseline outcome score and subsequently for age and sex. **Results**: Following the counseling intervention, statistically significant pre–post changes were observed in MG-ADL (*p* = 0.044), mBMI (*p* = 0.018), and PHQ-9 (*p* = 0.002) scores. After adjustment for the corresponding baseline outcome score, age, and sex, baseline depressive tendency was associated with less favorable post-counseling mBMI (*β* = −3.52, robust 95% CI −6.19 to −0.86, *p* = 0.014) and MG-ADL scores (*β* = 1.70, robust 95% CI 0.51 to 2.89, *p* = 0.009). **Conclusions**: Baseline depressive tendency was associated with poorer baseline functional and disease-specific quality-of-life status and with less favorable adjusted post-counseling MG-ADL and mBMI scores. These associations met the Benjamini–Hochberg threshold but not the more conservative Benjamini–Yekutieli threshold. Given the small sample and uncontrolled pilot design, the findings should not be regarded as evidence of counseling effectiveness and require confirmation in prospective controlled studies.

## 1. Introduction

Myasthenia gravis (MG) is a chronic autoimmune neuromuscular disorder characterized by fluctuating skeletal muscle weakness. The unpredictable and recurrent nature of the disease not only compromises patients’ physical functioning but also profoundly disrupts their lifestyle, self-identity, interpersonal relationships, and occupational functioning. Among the associated psychological burdens, depressive symptoms are particularly important because they represent one of the most prevalent emotional comorbidities in MG and are associated with symptom perception, treatment responsiveness, and overall quality of life [1].

A large cross-sectional study reported that approximately 31% of patients with MG experience clinically significant depressive symptoms, and psychological distress was strongly associated with MG-specific quality-of-life measures while also increasing caregiver burden [2]. Similarly, a Chinese study revealed that 22.5% of patients with thymoma experienced preoperative anxiety or depressive symptoms, with a higher prevalence observed among those with concomitant MG, highlighting the importance of routine psychological screening before surgery [3]. In addition, a South African study found that approximately 30% of patients with thymoma-associated MG exhibited moderate-to-severe depressive symptoms, and the severity of depression was closely associated with MG disease severity [4]. Collectively, these findings suggest that depressive tendency may substantially influence the clinical burden and disease experience of MG.

Recent studies have reported changes in psychological outcomes following participation in psychological interventions among patients with MG. An online mental health intervention study documented changes in psychological outcomes [5], whereas the MG-ADL scale has been used to assess daily functional status in psychosocial intervention research [6]. These findings reflect growing interest in incorporating mental health support into comprehensive MG care.

Psychological well-being has increasingly been recognized as a fundamental component of modern medical care. In 2009, the International Psycho-Oncology Society (IPOS) proposed psychological distress as the “sixth vital sign,” advocating routine screening and intervention in cancer care settings [7]. Similarly, the “social prescribing” model implemented in the United Kingdom integrates medical and community resources to provide non-pharmacological psychosocial support and has been shown to improve well-being, life satisfaction, and anxiety outcomes [8]. These developments collectively support the broader integration of psychosocial support into comprehensive disease management, while the clinical effects of specific counseling approaches require evaluation within individual patient populations.

Within the clinical context of MG, learned helplessness may provide one possible conceptual framework for understanding the interaction between depressive symptoms and functional dependency. The fluctuating and unpredictable muscle weakness characteristic of MG may impair patients’ independence in daily functioning and could contribute to a perceived loss of control over their physical condition. It is possible that, when functional dependency coexists with depressive symptoms, some patients may develop persistent negative beliefs regarding their capacity for improvement, which could in turn reduce engagement in treatment or counseling. This unmeasured and speculative mechanism may be particularly relevant to patients with thymoma-associated MG because surgical stress and postoperative immune instability could further contribute to perceived helplessness and dependency.

Accordingly, the primary research question was whether baseline depressive tendency was associated with post-counseling functional status, disease-specific quality of life, psychological well-being, and depressive symptoms after adjustment for the corresponding baseline outcome score, age, and sex. We hypothesized that participants with a baseline depressive tendency would exhibit a less favorable post-counseling clinical status. Given the exploratory pilot design, this hypothesis was intended to generate initial evidence for future confirmatory studies.

## 2. Materials and Methods

### 2.1. Study Design

Given the rarity of thymoma-associated MG, the limited pool of eligible patients who had undergone thymectomy, and the preliminary nature of the research question, a single-group pre–post pilot design was selected to generate initial estimates and inform the design of future controlled studies. Because this was an exploratory pilot study in a relatively uncommon clinical population, no formal a priori sample-size calculation based on a prespecified effect size was performed. The final sample size was determined by the number of eligible patients who agreed to participate and completed the study procedures during the recruitment period. This exploratory pilot study enrolled patients with thymoma-associated MG identified through the neurology outpatient clinics of Shin Kong Wu Ho-Su Memorial Hospital, Taipei, Taiwan. All eligible participants received eight sessions of structured psychological counseling. Structured questionnaires were administered at baseline and immediately after completion of the intervention to evaluate changes in psychological and clinical status.

Participants were classified into a depressive tendency group (PHQ-9 ≥ 10) and a non-depressive tendency group (PHQ-9 < 10) according to their baseline PHQ-9 scores. Baseline characteristics were compared between the two groups. In the regression analyses, depressive tendency was entered as a binary explanatory variable to examine its association with post-counseling MG-ADL, MG-QoL15, mBMI, and PHQ-9 scores after covariate adjustment.

The study period extended from 1 January 2024, to 31 December 2025, and consisted of four stages: (1) screening and baseline assessment; (2) completion of eight counseling sessions over approximately three months; (3) immediate post-intervention assessment; and (4) data processing and statistical analysis.

### 2.2. Participants

This study was approved by the Institutional Review Board of Shin Kong Wu Ho-Su Memorial Hospital, Taipei, Taiwan (IRB No. 20230730R; approved on 9 November 2023). Potentially eligible patients were screened by a case manager according to the prespecified eligibility criteria. Patients considered eligible were contacted either during outpatient visits or by telephone and were invited to participate in the study. After providing written informed consent, participants completed demographic information and baseline questionnaires through an online platform. Because recruitment relied on clinical screening and voluntary participation, the possibility of selection bias cannot be excluded.

**Figure 1:**
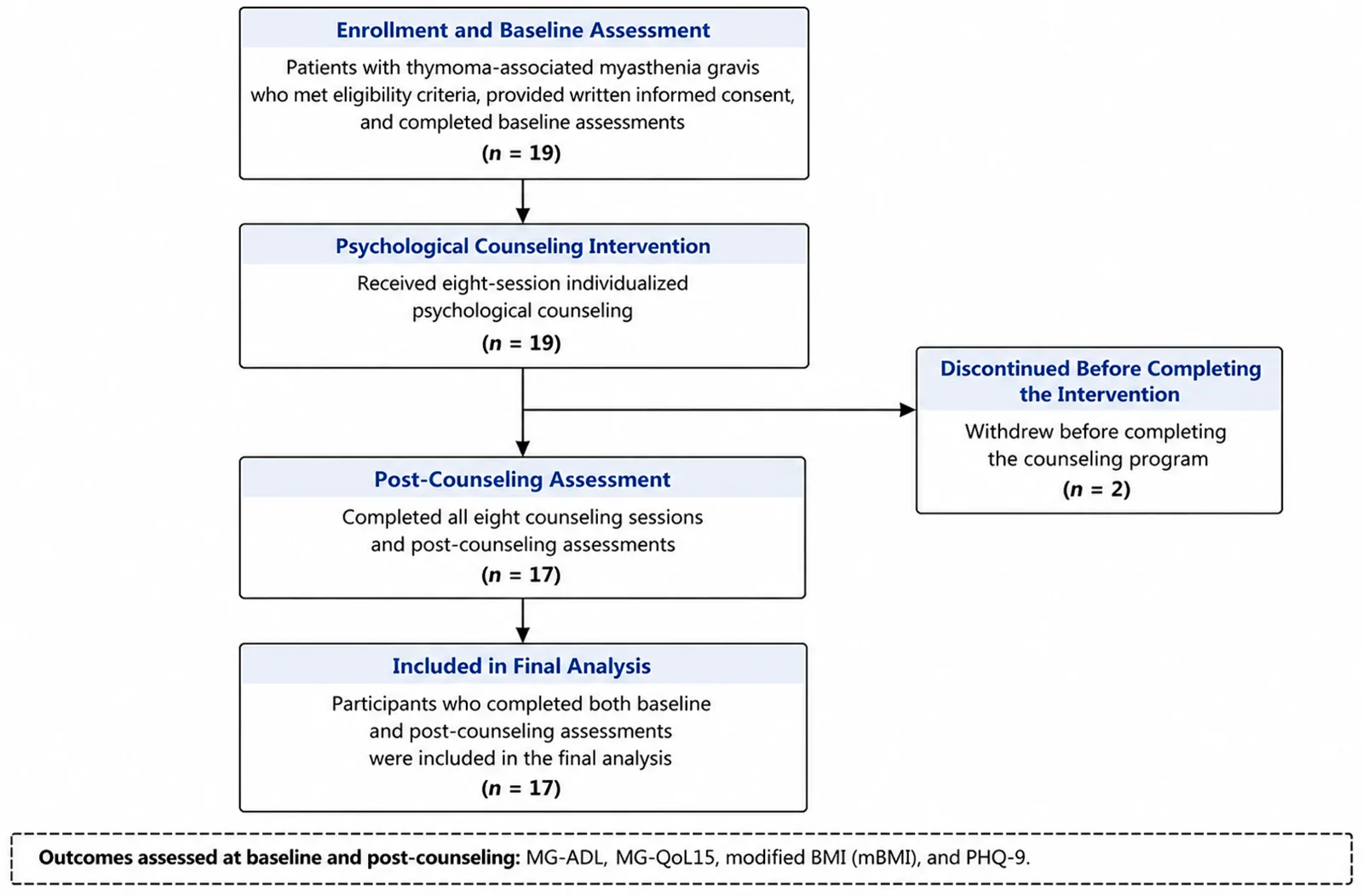
Flow diagram of participant enrollment, counseling completion, and final analysis. MG-ADL = Myasthenia Gravis Activities of Daily Living Scale; MG-QOL15 = Myasthenia Gravis Quality of Life 15-item; *n* = number of participants; PHQ-9 = Patient Health Ques-tionnaire-9.

Participant enrollment, counseling completion, and final analysis were presented as a flow diagram in Figure 1. A total of 19 participants completed baseline assessments during the study period. Two participants withdrew before completion of the intervention, and 17 participants completed both pre- and post-intervention assessments and were included in the final analysis.

The present study specifically enrolled patients diagnosed with thymoma-associated MG who had previously undergone thymectomy.

Participants were eligible for enrollment if they met the following criteria: (1) a confirmed diagnosis of MG associated with thymoma and prior completion of surgical treatment; (2) age ≥ 20 years; (3) ability to communicate clearly and provide reliable responses; (4) absence of cognitive impairment or severe psychiatric illness that could interfere with participation; and (5) willingness to participate in the study with provision of written informed consent.

Participants were excluded if they met any of the following criteria: (1) the absence of a confirmed diagnosis of myasthenia gravis; (2) failure to confirm thymoma on postoperative pathological examination; (3) inability to communicate adequately in Mandarin, Taiwanese, English, or written form; or (4) the presence of conditions that could compromise verbal communication or regular participation in counseling sessions, such as significant traumatic brain injury, severe psychiatric disorders, or cognitive impairment.

### 2.3. Intervention

The psychological counseling intervention was based primarily on Gestalt therapy principles, emphasizing awareness and expression of suppressed emotions as well as resolution of unfinished emotional experiences [9]. The intervention additionally incorporated Somatic Experiencing techniques, which focused on bodily awareness and tension release to facilitate emotional regulation and psychological well-being.

Each participant received eight individualized face-to-face counseling sessions delivered weekly or biweekly. Each session lasted approximately 60 min. All sessions were conducted by licensed counseling psychologists in a private and secure consultation setting. No audio or video recording was performed, and session records were encrypted and securely stored after completion.

The counseling intervention was conducted according to a structured therapeutic framework consisting of three phases: the initial phase, working phase, and termination phase (Table 1). Considering individual differences among participants, the detailed content and pacing of each session could be adjusted as clinically appropriate; however, the overall therapeutic structure remained consistent throughout the intervention.

### 2.4. Definition of Depressive Tendency

Baseline depressive severity was assessed using the Patient Health Questionnaire-9 (PHQ-9), which served as the operational definition of depressive tendency in this study. The PHQ-9 is a nine-item self-administered questionnaire designed to evaluate the frequency of depression-related symptoms experienced during the preceding two weeks. Total scores range from 0 to 27, with higher scores indicating greater severity of depressive symptoms. Depression severity can be categorized as minimal (1–4), mild (5–9), moderate (10–14), moderately severe (15–19), and severe (20–27). Higher PHQ-9 scores are associated with greater predictive value and likelihood ratios for major depressive disorder.

The present study adopted the Chinese version of the PHQ-9 developed by Liu et al. (2011) [10], which reported good psychometric properties, with a Cronbach’s α of 0.80 and test–retest reliability of 0.87. A cutoff score of PHQ-9 ≥ 10 yielded a sensitivity of 0.86 and a specificity of 0.94 for DSM-IV major depressive disorder. Based on this validated clinical cutoff, participants with baseline PHQ-9 scores ≥ 10 were classified into the depressive tendency group, whereas those with scores < 10 were classified into the non-depressive tendency group. This binary classification was used as the principal explanatory variable in group comparisons and multivariable regression analyses of post-counseling clinical status. Because the PHQ-9 is a screening instrument rather than a structured diagnostic interview, this classification should be interpreted as indicating depressive tendency and not as a formal diagnosis of major depressive disorder.

### 2.5. Structural Questionnaires—Clinical Indices/Measures

Three structured questionnaires were used as the primary instruments to evaluate clinical changes before and after the counseling intervention. These measures assessed patients’ daily functional status, disease-specific quality of life, and psychological well-being.

#### 2.5.1. Myasthenia Gravis Activities of Daily Living Scale (MG-ADL)

The Myasthenia Gravis Activities of Daily Living scale (MG-ADL) was developed by Wolfe et al. (1999) [11] based on modifications of the Quantitative Myasthenia Gravis (QMG) score proposed by Tindall et al. The MG-ADL is designed to assess daily functional impairment in patients with MG.

The scale consists of eight items covering ocular function (two items: diplopia and ptosis), bulbar function (three items: speech, chewing, and swallowing), respiratory function (one item), and limb muscle function (two items: personal hygiene and ability to rise from a chair). Each item is scored on a 4-point scale ranging from 0 (normal) to 3 (most severe), yielding a total score ranging from 0 to 24. Higher scores indicate greater functional impairment. The MG-ADL has demonstrated good convergent validity with the QMG score, with a Pearson correlation coefficient of 0.583 (*p* < 0.001) [11].

#### 2.5.2. Myasthenia Gravis Quality of Life Scale (MG-QoL15)

The Myasthenia Gravis Quality of Life 15-item scale (MG-QoL15) was developed by Burns et al. (2008) [12] as a shortened version of the original 60-item instrument proposed by Mullins et al. [13]. The MG-QoL15 consists of 15 items encompassing four domains: mobility (9 items), symptoms (3 items), emotional well-being (2 items), and general contentment (1 item).

Each item is rated on a 5-point Likert scale ranging from 0 (“not at all”) to 4 (“very much”). Higher total scores indicate a greater negative impact of MG on quality of life. The MG-QoL15 has reported good psychometric performance, with a Cronbach’s α of 0.89, indicating satisfactory reliability and validity [12].

#### 2.5.3. Mental BMI (mBMI)

The Mental BMI (mBMI) psychological well-being index was developed by Professor Li, Y.C. (2016) [14] and is based on three operational psychological health skills: Befriend (B), Mindfulness (M), and Identity (I). Each domain is assessed using a single self-rated item scored from 0 to 10, with higher total scores indicating better psychological well-being. A total score ≥ 20.5 is considered indicative of favorable mental health status.

Previous studies have shown that both the total mBMI score and its individual components effectively predict social, emotional, and psychological well-being measured by the Mental Health Continuum–Short Form, with a total explanatory power of 30.3% [15]. Confirmatory factor analysis demonstrated acceptable psychometric properties, including a composite reliability (CR) of 0.80 and an average variance extracted (AVE) of 0.56, supporting its construct validity and discriminative ability. Accordingly, the mBMI is considered a practical and concise instrument for evaluating psychological health in intervention studies.

### 2.6. Covariates

Demographic covariates included sex, age, educational level, marital status, living arrangement, participation in social groups, employment status, and monthly income.

Sex was categorized as male or female. Age was collected in three prespecified categories (18–44, 45–64, and ≥65 years) and was entered into the primary regression models as an ordinal variable coded 1, 2, and 3, respectively. Because exact age in years was not collected, analysis using continuous chronological age was not possible. As a sensitivity analysis, age group was additionally modeled using categorical indicator variables. Educational level was categorized into junior high school or below, senior high school, and college or above. Marital status was categorized as unmarried, married, or divorced/widowed. Living arrangement was categorized as living alone, living with family members, residing in a care institution, or other. Participation in social groups was classified as participation or no participation. Employment status included full-time employment, part-time employment, unpaid leave, retirement, and unemployment. Monthly income was categorized as no income, ≤NT$30,000, or >NT$30,000.

### 2.7. Statistical Analysis

All analyses were performed using Stata version 18.0, with two-sided *p* values < 0.05 considered statistically significant. Continuous variables are presented as means and standard deviations, and categorical variables as counts and percentages. Paired-sample t-tests were used to compare pre- and post-counseling MG-ADL, MG-QoL15, mBMI, and PHQ-9 scores. Within-participant effect sizes were expressed as Cohen’s dz, calculated as the mean paired difference divided by the standard deviation of the paired differences.

Separate linear regression models were constructed for each post-counseling outcome. Baseline depressive tendency was entered as the primary explanatory variable. The crude model included depressive tendency alone; Model 1 additionally adjusted for the corresponding baseline outcome score; and Model 2 further adjusted for age and sex. Regression coefficients are reported with 95% confidence intervals.

For the paired analyses, the distributions of the change scores were evaluated visually and using the Shapiro–Wilk test. Wilcoxon signed-rank tests were conducted as sensitivity analyses. For the fully adjusted regression models, linearity and residual patterns were visually assessed using residual-versus-fitted plots; residual normality was evaluated using the Shapiro–Wilk test; homoscedasticity using the Breusch–Pagan/Cook–Weisberg test; and multicollinearity using variance inflation factors (VIFs). Individual and mean VIFs were calculated for each fully adjusted outcome model and are presented in Supplementary Table S1. Because of the small sample size, these diagnostic tests were interpreted cautiously. Leave-one-out sensitivity analyses were also conducted for the principal MG-ADL and mBMI regression estimates. Because the Breusch–Pagan/Cook–Weisberg test indicated heteroskedasticity in the mBMI model, heteroskedasticity-robust standard errors were applied to the fully adjusted regression models. Robust standard errors were reported for all Model 2 estimates to provide a consistent inferential approach across outcomes.

Because baseline depressive tendency was defined directly from the continuous baseline PHQ-9 score using a cutoff of ≥10, simultaneous inclusion of both variables in the fully adjusted PHQ-9 model could reduce the precision and independent interpretability of their individual coefficients. Therefore, two post hoc PHQ-9-specific sensitivity models were fitted using alternative, non-overlapping representations of baseline depression. Sensitivity Model A included baseline depressive tendency, age group, and sex but excluded the continuous baseline PHQ-9 score. Sensitivity Model B included the continuous baseline PHQ-9 score, age group, and sex but excluded the binary depressive-tendency indicator. Heteroskedasticity-robust standard errors were used for both sensitivity models. These analyses were undertaken to evaluate sensitivity to covariate specification rather than to replace the prespecified fully adjusted model or to seek statistical significance. The results are presented in Supplementary Table S2.

No multiplicity adjustment was prespecified because the analyses were exploratory. As a post hoc sensitivity analysis, the four heteroskedasticity-robust p values from the fully adjusted Model 2 analyses, representing four parallel post-counseling outcomes, were adjusted using the Benjamini–Hochberg false discovery rate procedure. Because the outcomes were measured in the same participants and their dependence structure could not be fully characterized, the more conservative Benjamini–Yekutieli procedure was additionally applied as a sensitivity analysis. Multiplicity-adjusted results were regarded as supplementary robustness analyses rather than confirmatory inference.

## 3. Results

### 3.1. Baseline Clinical Characteristics of Thymoma-Associated MG Patients Stratified by Depressive Tendency

During the study period, 19 participants were enrolled and completed baseline assessment. Of these, 17 completed both the baseline and post-counseling assessments and were included in the final analysis, whereas two withdrew before completing the counseling program. The overall cohort consisted predominantly of women (*n* = 13, 76.5%), and most participants were between 45 and 65 years of age (*n* = 11, 64.7%) (Table 2).

Participants were stratified according to baseline PHQ-9 scores using a cutoff value of 10 into a non-depressive tendency group (PHQ-9 < 10, *n* = 11, 64.7%) and a depressive tendency group (PHQ-9 ≥ 10, *n* = 6, 35.3%). Detailed baseline clinical characteristics are presented in Table 2.

No significant differences were observed between groups in age distribution (*p* = 0.760) or sex (*p* = 0.584). However, participants with depressive tendency had less favorable baseline disease-related clinical status. The mean MG-ADL score was significantly higher in the depressive tendency group than in the non-depressive tendency group (3.83 ± 2.3 vs. 1.73 ± 1.5, *p* = 0.037), indicating greater impairment in daily functioning. Likewise, the mean MG-QoL15 score was significantly higher in the depressive tendency group (14.50 ± 7.1 vs. 4.00 ± 3.4, *p* < 0.001), suggesting a greater negative impact of the disease on quality of life.

Although mBMI scores did not reach statistical significance, the depressive tendency group showed a trend toward lower scores compared with the non-depressive tendency group (18.50 ± 5.5 vs. 23.09 ± 3.1, *p* = 0.063), indicating poorer psychological well-being. As expected, PHQ-9 scores differed significantly between groups due to the predefined cutoff criterion, with mean scores of 11.33 ± 2.1 and 4.73 ± 2.2, respectively (*p* < 0.001).

These findings indicate that patients with depressive tendency entered counseling with poorer baseline functional status and disease-specific quality of life, suggesting greater baseline clinical burden in this subgroup.

### 3.2. Pre–Post Clinical Changes and Associations Between Baseline Depressive Tendency and Post-Counseling Clinical Status

Following eight sessions of structured psychological counseling, overall MG-ADL scores significantly decreased (Δ = −0.82, *p* = 0.044), indicating improvement in daily functional status. In addition, mBMI scores significantly increased (Δ = +2.41, *p* = 0.018), reflecting improved psychological well-being. Although MG-QoL15 scores showed a decreasing trend after intervention (Δ = −0.82), the change did not reach statistical significance (*p* = 0.576). The markedly increased post-counseling standard deviation (SD = 10.89) suggested substantial interindividual variability in MG-QoL15 scores. PHQ-9 scores also decreased significantly from 7.06 ± 3.88 to 4.59 ± 4.08 (mean difference = −2.47, p = 0.002). Overall, statistically significant pre–post changes were observed in MG-ADL, mBMI, and PHQ-9 scores, whereas MG-QoL15 scores did not change significantly (Table 3).

To examine whether baseline depressive tendency was associated with post-counseling clinical status, separate linear regression models were fitted using all 17 participants. Baseline depressive tendency was entered as the primary binary explanatory variable. The crude model included depressive tendency alone; Model 1 additionally adjusted for the corresponding baseline outcome score; and Model 2 further adjusted for age and sex (Table 4). Participants were not analyzed in separate subgroup-specific regression models. Table 2 presents unadjusted between-group differences in baseline characteristics, whereas Table 4 presents differences in post-counseling outcomes associated with baseline depressive tendency after sequential adjustment for the corresponding baseline outcome score, age group, and sex.

In the fully adjusted models using heteroskedasticity-robust standard errors, participants with baseline depressive tendency had post-counseling mBMI scores that were, on average, 3.52 points lower than those without depressive tendency (*β* = −3.52, robust 95% CI −6.19 to −0.86, *p* = 0.014). The incremental *R*^2^ attributable to baseline depressive tendency was 0.124.

Similarly, participants with baseline depressive tendency had post-counseling MG-ADL scores that were, on average, 1.70 points higher than those without depressive tendency after adjustment for baseline MG-ADL, age group, and sex (*β* = 1.70, robust 95% CI 0.51 to 2.89, *p* = 0.009). The incremental *R*^2^ attributable to baseline depressive tendency was 0.130.

No statistically significant adjusted association was observed for MG-QoL15 (*β* = 1.77, robust 95% CI −9.32 to 12.87, *p* = 0.733).

For the PHQ-9 outcome, the adjusted coefficient for baseline depressive tendency was not statistically significant (*β* = 3.62, robust 95% CI −1.08 to 8.32, *p* = 0.119). In this model, the VIFs for baseline depressive tendency and continuous baseline PHQ-9 were 4.00 and 3.68, respectively, whereas the VIFs for age group and sex were 1.18 and 1.20. Although all VIFs were below 5, the comparatively elevated values for the two baseline depression variables were expected because the binary depressive-tendency indicator was derived directly from the continuous baseline PHQ-9 score. Combined with the small sample size, this structural overlap limited the precision and independent interpretability of the individual depressive-tendency coefficient. The PHQ-9 result was therefore considered inconclusive under the current covariate structure rather than evidence of no association. Complete VIF results for all four fully adjusted outcome models are provided in Supplementary Table S1.

In PHQ-9-specific sensitivity analyses using alternative covariate specifications, baseline depressive tendency was positively associated with post-counseling PHQ-9 when the continuous baseline PHQ-9 score was excluded (Sensitivity Model A: *β* = 6.20, robust 95% CI 3.36 to 9.05, *p* < 0.001; *R*^2^ = 0.739). When the binary depressive-tendency indicator was excluded, continuous baseline PHQ-9 was also positively associated with post-counseling PHQ-9 (Sensitivity Model B: *β* = 0.74 per one-point higher baseline PHQ-9 score, robust 95% CI 0.48 to 1.01, *p* < 0.001; *R*^2^ = 0.725). VIFs were low in both alternative models. These findings indicate that the PHQ-9 estimates were sensitive to the representation of baseline depression and support cautious interpretation of the individual depressive-tendency coefficient in the original fully adjusted model. Full sensitivity-analysis results are shown in Supplementary Table S2.

In leave-one-out sensitivity analyses, the MG-ADL coefficient remained positive in all 17 iterations (range, 1.46–2.14), whereas the mBMI coefficient remained negative in all iterations (range, −4.66 to −2.67), indicating that the directions of the estimates were not driven by a single participant. However, statistical significance was retained in only 13 of 17 MG-ADL iterations and 9 of 17 mBMI iterations, demonstrating that the p values—particularly for mBMI—were sensitive to the composition of this small sample.

Sensitivity analyses treating age group as a categorical variable yielded coefficients of similar direction and magnitude. The MG-ADL and mBMI associations remained statistically significant, whereas the MG-QoL15 coefficient remained nonsignificant. The PHQ-9 depressive-tendency coefficient also remained nonsignificant in this alternative age specification; however, its interpretation remained limited by the simultaneous inclusion of the binary depressive-tendency indicator and continuous baseline PHQ-9 score.

In a post hoc multiplicity sensitivity analysis of the four fully adjusted Model 2 outcomes, Benjamini–Hochberg adjustment of the heteroskedasticity-robust *p* values yielded *q* = 0.028 for both MG-ADL and mBMI, whereas the adjusted q values for PHQ-9 and MG-QoL15 were 0.159 and 0.733, respectively. Thus, the MG-ADL and mBMI associations remained statistically significant at a false discovery rate of 5% under the Benjamini–Hochberg procedure. Under the more conservative Benjamini–Yekutieli procedure, the corresponding *q* values for MG-ADL and mBMI were both 0.058 and therefore did not meet the 0.05 threshold. These multiplicity analyses were treated as sensitivity analyses and did not materially alter the overall pattern of results.

Collectively, these findings suggest that baseline depressive tendency was associated with post-counseling MG-ADL and mBMI scores after covariate adjustment. For PHQ-9, the primary fully adjusted depressive-tendency coefficient was inconclusive under the original covariate structure, while alternative specifications indicated that post-counseling PHQ-9 remained associated with the baseline depression measure retained in each model. Given the small sample size, sensitivity to individual observations, covariate-specification dependence, and pilot-study analytical framework, all adjusted findings should be interpreted with appropriate caution.

## 4. Discussion

This exploratory pilot study examined pre–post clinical changes during an eight-session counseling period and the association between baseline depressive tendency and post-counseling clinical status in patients with thymoma-associated MG. Two principal findings emerged. First, statistically significant pre–post changes were observed in MG-ADL, mBMI, and PHQ-9 scores, whereas MG-QoL15 did not change significantly. Second, after adjustment for the corresponding baseline outcome score, age group, and sex, baseline depressive tendency was associated with less favorable post-counseling MG-ADL and mBMI scores. For PHQ-9, however, simultaneous adjustment for the binary depressive-tendency indicator and the continuous score from which it was derived limited the independent interpretability of the primary coefficient; alternative covariate specifications demonstrated that the PHQ-9 findings were sensitive to how baseline depression was represented. The MG-ADL and mBMI associations remained statistically significant when heteroskedasticity-robust standard errors were applied, when age group was alternatively modeled using categorical indicator variables, and after Benjamini–Hochberg adjustment of the four fully adjusted outcome models. However, under the more conservative Benjamini–Yekutieli procedure, neither association met the 0.05 threshold (both *q* = 0.058). Together with the sensitivity of statistical significance to individual observations in leave-one-out analyses, these findings should therefore be regarded as exploratory and warrant confirmation in larger controlled studies. Although formal feasibility outcomes were not prespecified, completion of the eight-session counseling program and paired assessments by 17 of the 19 participants who completed baseline assessment provides descriptive information that may assist future study planning.

The prevalence of depressive tendency in the present cohort was 35.3% (PHQ-9 ≥10), which is consistent with previous epidemiological reports. Freeman et al. (2014) [4] reported that approximately 30% of patients with thymoma-associated MG exhibited moderate-to-severe depressive symptoms, whereas Marbin et al. (2022) [2] found that approximately 31% of MG patients experienced clinically significant depression, which was strongly correlated with MG-specific quality of life. The prevalence observed in our study therefore aligns closely with existing literature and further reinforces the substantial psychological burden among patients with thymoma-associated MG.

Furthermore, patients with depressive tendencies had significantly worse baseline MG-ADL and MG-QoL15 scores, suggesting a greater functional and psychosocial burden. These findings are concordant with previous reports [2] indicating that depressive symptoms amplify functional impairment and reduce quality of life in patients with MG.

Regarding the overall findings of this study, the observed reductions in MG-ADL scores and increases in mBMI scores were broadly consistent with previous reports suggesting a potential association between psychological interventions and mental health outcomes in patients with MG [5]. While the present study was not designed to establish causal effects, these findings suggest that participation in a structured face-to-face Gestalt-oriented counseling program coincided with changes in functional status, depressive symptoms, and psychological well-being. Taken together, these observations extend the limited literature and provide initial evidence to inform future controlled studies.

The integrative therapeutic approach adopted in this study combined Gestalt therapy with Somatic Experiencing techniques, emphasizing direct emotional awareness and expression, resolution of unfinished emotional experiences, and emotional regulation through bodily awareness and tension release. By simultaneously addressing emotional and physiological dimensions of distress, this integrative model may help address the complex interaction between neuromuscular symptoms and psychological stress in patients with MG. Although changes in MG-ADL and mBMI were observed during the counseling period, the present uncontrolled design cannot determine whether these changes were attributable to the proposed therapeutic mechanisms.

In contrast, MG-QoL15 scores did not show a statistically significant pre–post change (*p* = 0.576), and the larger post-counseling standard deviation indicated substantial interindividual variability. MG-QoL15 was included because it is a well-validated disease-specific measure that captures mobility, symptom burden, emotional well-being, and general contentment. Accordingly, the null finding should be considered a substantive result rather than a limitation of the instrument. One possible interpretation is that MG-specific quality of life may be more strongly influenced by neuromuscular disease activity, functional impairment, and medical management than by short-term psychological counseling alone [16,17,18,19]; however, this explanation remains speculative.

From a clinical perspective, the principal exploratory finding was that baseline depressive tendency was associated with less favorable post-counseling MG-ADL and mBMI scores after adjustment for the corresponding baseline scores, age, and sex. Specifically, participants with depressive tendency had adjusted post-counseling MG-ADL scores 1.70 points higher and mBMI scores 3.52 points lower than those without depressive tendency. These adjusted differences indicate that participants with baseline depressive tendency exhibited less favorable short-term functional and psychological status after counseling. However, the small sample size, model instability, and absence of a control group preclude causal or definitive predictive interpretation.

In terms of risk stratification, baseline depressive tendency may warrant further study as a potential marker of clinically relevant post-counseling differences. In contrast, the corresponding baseline outcome score, age group, and sex were included as adjustment covariates and were not evaluated as established risk or protective factors. No specific protective factor was identified in the present study. The adjusted MG-ADL difference of 1.70 points and mBMI difference of −3.52 points provide information regarding the magnitude of the observed associations; however, these estimates represent differences in absolute post-counseling status rather than differential counseling benefit. Furthermore, because a validated minimal clinically important difference for mBMI was not applied, the clinical relevance of the mBMI estimate remains uncertain. These findings should therefore be regarded as supporting potential risk stratification and hypothesis generation rather than definitive clinical prediction.

One possible conceptual explanation for these findings is the framework of depressive dependency, which can be conceptualized as an integration of interpersonal dependency, sociotropic vulnerability, and learned helplessness. Previous studies have demonstrated that dependency-related personality traits are associated with depressive symptoms, helplessness, maladaptive self-beliefs, and differential responses to psychotherapy [20,21,22], suggesting that patients who perceive themselves as unable to influence their circumstances may be less likely to actively engage in therapeutic processes. The fluctuating muscle weakness characteristic of MG may progressively impair patients’ autonomy in daily functioning, thereby fostering a state of learned helplessness. When such functional dependency further interacts synergistically with depressive emotions, patients may develop maladaptive cognitive beliefs that their condition is “unchangeable,” consequently reducing both their motivation and capacity to actively engage in the counseling process. This speculative framework may offer one possible interpretation of why patients with baseline depressive tendency exhibited less favorable adjusted MG-ADL and mBMI status after counseling; however, the proposed mechanism was not directly measured in this study.

For patients with thymoma-associated MG, the additional physical burden imposed by surgery and postoperative immune fluctuations may further exacerbate this maladaptive psychosomatic cycle, as thymectomy has been shown to induce transient cytokine alterations, long-term immune modulation, and heterogeneous postoperative neurological responses in MG patients [23,24].

Moreover, the present findings are broadly consistent with the overall trend observed in psychological intervention research for chronic illnesses, in which greater baseline depressive severity has been shown to predict differential responsiveness to psychotherapy and psychosocial intervention outcomes [25]. This conceptual alignment offers additional theoretical context for understanding the possible relationship between depressive tendency and psychosocial intervention responsiveness in thymoma-associated MG.

Baseline depressive tendency was not significantly associated with post-counseling MG-QoL15 scores in the crude or adjusted models. Together with the nonsignificant pre–post change, this finding provides no statistical evidence in the present sample that depressive tendency was associated with short-term MG-specific quality-of-life outcomes.

The PHQ-9 outcome requires a distinct interpretation. In the primary fully adjusted model, baseline depressive tendency was entered together with continuous baseline PHQ-9, even though the binary indicator was derived directly from the continuous score. The resulting VIFs were elevated relative to those in the other outcome models, although they remained below 5. In conjunction with the small sample size, this structural overlap reduced the precision with which the independent coefficient for depressive tendency could be estimated. Accordingly, its nonsignificant coefficient should not be interpreted as evidence that baseline depressive tendency was unrelated to post-counseling depressive symptoms. In sensitivity analyses that retained only one representation of baseline depression, both the binary depressive-tendency indicator and continuous baseline PHQ-9 were positively associated with post-counseling PHQ-9 in their respective models. These results indicate sensitivity to covariate specification and support characterizing the primary PHQ-9 estimate as inconclusive rather than null. Nevertheless, neither alternative model establishes a causal effect or differential counseling response, and the findings require confirmation in a substantially larger sample.

Several limitations should be acknowledged. First, the final sample size was small (*n* = 17), limiting statistical power and increasing susceptibility to both imprecise estimates and type II error. The fully adjusted regression models included four explanatory variables in only 17 participants, resulting in a low subjects-per-variable ratio and a substantial risk of overfitting and unstable statistical inference. Moreover, only six participants were classified as having baseline depressive tendency, and these participants were distributed across three age categories. The resulting sparse age-by-depressive-tendency cells further limited the stability and interpretability of the age-adjusted estimates, irrespective of whether age group was modeled as an ordinal or categorical variable. Although leave-one-out analyses showed that the directions of the MG-ADL and mBMI coefficients remained consistent, the statistical significance of these estimates—particularly for mBMI—was sensitive to the removal of individual observations. In addition, no multiplicity adjustment was prespecified, and multiple outcomes and model specifications were examined. In a post hoc sensitivity analysis of the four fully adjusted outcome models, the MG-ADL and mBMI associations remained significant after Benjamini–Hochberg adjustment (both *q* = 0.028) but did not meet the 0.05 threshold under the more conservative Benjamini–Yekutieli procedure (both *q* = 0.058). This dependence on the multiplicity procedure further underscores the pilot-phase nature of the findings and the need for confirmation in an adequately powered study with prespecified primary outcomes. Formal regression diagnostic tests also have limited power in a sample of this size. In the PHQ-9 model, the binary depressive-tendency indicator and the continuous baseline PHQ-9 score were structurally related, resulting in comparatively elevated VIFs and reduced precision for the individual depressive-tendency coefficient. Although post hoc sensitivity models using alternative representations of baseline depression were performed, these analyses were not prespecified and should be interpreted as specification checks rather than independent confirmatory tests. Accordingly, the adjusted findings should be considered exploratory and hypothesis-generating. In addition, depressive tendency was operationally defined using a binary PHQ-9 cutoff of ≥10. Although this threshold was based on the validated Chinese version of the PHQ-9, dichotomization may introduce classification uncertainty, particularly for participants with scores close to the cutoff, and may reduce information regarding the underlying continuum of depressive symptom severity. This concern is particularly relevant in the present small sample, in which only six participants met the criterion for depressive tendency; reclassification of a single participant could potentially influence estimates based on the binary explanatory variable. Future adequately powered studies should examine depressive symptoms as a continuous measure and assess the robustness of findings across alternative clinically relevant thresholds. Second, the single-group pre–post design lacked a control group, precluding causal inference and leaving open alternative explanations such as spontaneous disease fluctuation, regression to the mean, maturation, Hawthorne or expectancy effects, and concurrent changes in medical treatment. Third, post-counseling assessments were conducted immediately after completion of counseling, without long-term follow-up; therefore, the durability of the observed changes could not be evaluated. Fourth, several potentially important clinical and psychosocial confounders—including disease duration, medication regimen or intensity, treatment changes during follow-up, objective neuromuscular severity, social support, and stressful life events—were not incorporated into the analyses. Residual confounding from these unmeasured or unmodeled variables may therefore have influenced the observed outcomes. Fifth, the psychometric evidence supporting the mBMI remains limited by conventional international standards because the instrument has primarily been described in a Chinese-language professional publication and a master’s thesis rather than extensively validated in international peer-reviewed populations. Although preliminary reliability and construct-validity findings have been reported, the measurement properties and minimal clinically important difference in the mBMI require further independent validation. Sixth, qualitative data were not systematically collected; therefore, participants’ subjective experiences, engagement with counseling, and potential mechanisms underlying the observed changes could not be explored. Future mixed-methods studies may provide complementary insight into these processes. Finally, all counseling sessions were delivered by licensed psychologists from a single institution, and therapist effects cannot be fully excluded.

Regarding external validity, this single-center study included a sample composed predominantly of women (76.5%) and adults aged 45–64 years (64.7%). In addition, only patients with thymoma-associated MG who had undergone thymectomy were enrolled. The generalizability of the findings should therefore be limited to similar clinical settings, and the results should not be directly extrapolated to other MG subtypes or broader neuromuscular disease populations.

Future research should prioritize several directions. Randomized controlled trials—or, at minimum, studies incorporating waitlist control groups—should be considered to strengthen causal inference. Follow-up periods of at least six months are also needed to evaluate the durability of observed changes and longer-term clinical outcomes.

Future comparative studies may also evaluate other evidence-based psychotherapeutic approaches, such as cognitive behavioral therapy, in head-to-head designs. Such studies could clarify whether different therapeutic approaches are associated with differential outcomes according to baseline depressive tendency or clinical status.

Measurement could be strengthened by incorporating objective indicators of disease severity, such as acetylcholine receptor antibody titers and Quantitative Myasthenia Gravis (QMG) scores, into multi-variable models. This approach may help distinguish the relative contributions of disease activity and psychological factors to post-counseling outcomes. In larger samples, stratified or interaction analyses by age and disease severity may further clarify whether the association between depressive tendency and post-counseling clinical status varies across clinically relevant subgroups.

From a clinical perspective, the present preliminary findings suggest that baseline depressive tendency may warrant further investigation as a potential factor associated with post-counseling clinical status in patients with thymoma-associated MG. Whether routine PHQ-9 screening before psychological counseling has clinical utility remains uncertain and should be evaluated in larger prospective controlled studies.

## 5. Conclusions

In this exploratory pilot study of patients with thymoma-associated myasthenia gravis following thymectomy, statistically significant pre–post changes in MG-ADL, mBMI, and PHQ-9 scores were observed during an eight-session counseling period, whereas MG-QoL15 scores did not change significantly. Because the study lacked a control group, these changes cannot be attributed causally to the counseling intervention.

After adjustment for the corresponding baseline outcome score, age, and sex, baseline depressive tendency was associated with less favorable post-counseling MG-ADL and mBMI scores. However, these associations were estimated from a small sample, were sensitive to individual observations, and were examined within an exploratory multiple-outcome framework. They should therefore be interpreted as hypothesis-generating rather than as evidence that depressive tendency definitively predicts or limits counseling effectiveness.

The findings suggest that baseline depression screening may warrant further investigation in multidisciplinary care for thymoma-associated MG. Larger multicenter studies with appropriate control groups, prespecified primary outcomes, longer follow-up, and more comprehensive clinical covariates are required to confirm these preliminary associations and determine their clinical relevance.

## Figures and Tables

**Table 1:** Structured Framework of the Counseling Intervention.

Phase	Sessions	Counseling Content
Initial Phase	1–2	Establishment of the therapeutic relationship; assessment of participants’ physical and psychological conditions; identification of participants’ current difficulties and challenges
Working Phase	3–6	Clarification of participants’ external environmental stressors; exploration and expression of internal emotional experiences; resolution of suppressed unfinished emotional issues; focus on bodily sensations through body-awareness and tension-release exercises; cultivation of mindfulness, present-moment awareness, and self-care practices
Termination Phase	7–8	Review of the counseling process; highlighting participants’ growth and therapeutic gains; enhancement of self-efficacy; termination of the therapeutic relationship and provision of supportive closure

**Table 2:** Baseline clinical characteristics of patients with thymoma-associated myasthenia gravis stratified by baseline depressive tendency.

		Baseline Depressive Severity		
	Total	Non-Depressive Tendency	Depressive Tendency	
	*n* = 17	*n* = 11 (64.7%)	*n* = 6 (35.3%)	*p*-Value
**Age group**				0.760
18–44 y/o	3 (17.65)	2 (18.2)	1 (16.7)	
45–64 y/o	11 (64.71)	8 (72.7)	3 (50.0)	
≥65 y/o	3 (17.65)	1 (9.1)	2 (33.3)	
**Sex**				
Female	13 (76.5)	9 (81.8)	4 (66.7%)	0.584
**Baseline Scores (mean ± SD**)				
MG-ADL	2.47 ± 2.0	1.73 ± 1.5	3.83 ± 2.3	0.037
MG-QoL15	7.71 ± 7.1	4 ± 3.4	14.5 ± 7.1	<0.001
mBMI	21.47 ± 4.9	23.09 ± 3.1	18.5 ± 5.5	0.063
PHQ-9	7.06 ± 3.9	4.73 ± 2.2	11.33 ± 2.1	<0.001

SD = standard deviation; MG-ADL = Myasthenia Gravis Activities of Daily Living Scale; MG-QOL15 = Myasthenia Gravis Quality of Life 15-item; *n* = number of participants; PHQ-9 = Patient Health Ques-tionnaire-9.

**Table 3:** Pre–post clinical changes during the counseling period in patients with thymoma-associated myasthenia gravis.

	At Baseline	Post-Counseling				
	Mean ± SD	Mean ± SD	Mean Difference (Post–Pre)	95% CI for the Mean Difference	Cohen’s dz	*p*-Value
MG-ADL	2.47 ± 2.03	1.65 ± 1.84	–0.82	−1.62 to −0.03	−0.53	0.0437
MG-QoL15	7.71 ± 7.05	6.88 ± 10.89	–0.82	−3.88 to 2.23	−0.14	0.5755
mBMI	21.47 ± 4.91	23.88 ± 4.12	2.41	0.48 to 4.34	+0.64	0.0176
PHQ-9	7.06 ± 3.88	4.59 ± 4.08	–2.47	−3.91 to −1.03	−0.88	0.0023

SD, standard deviation; CI, confidence interval; MG-ADL, Myasthenia Gravis Activities of Daily Living scale; MG-QoL15, Myasthenia Gravis Quality of Life 15-item scale; mBMI, mental BMI; PHQ-9, Patient Health Questionnaire-9. Mean differences were calculated as post-counseling minus baseline scores. Cohen’s dz was calculated as the mean paired difference divided by the standard deviation of the paired differences. Negative values indicate decreases from baseline, whereas positive values indicate increases.

**Table 4:** Associations between baseline depressive tendency and post-counseling clinical outcomes in patients with thymoma-associated myasthenia gravis.

	Baseline Depressive Severity			
	Coef.	[95% CI]	*p*-Value	Incremental *R*^2^
**MG-ADL**				
Crude	2.35	(0.76, 3.94)	0.007	-
model 1	1.42	(–0.20, 3.04)	0.081	-
model 2	1.70	(0.51, 2.89)	0.009	0.130
**MG-QoL** **15**				
Crude	14.09	(4.72, 23.46)	0.006	-
model 1	0.12	(−9.20, 9.44)	0.978	-
model 2	1.77	(–9.32, 12.87)	0.733	0.003
**mBMI**				
Crude	–6.00	(−9.21, −2.79)	0.001	-
model 1	–4.36	(−7.51, −1.20)	0.010	-
model 2	–3.52	(−6.19, −0.86)	0.014	0.124
**PHQ-9**				
Crude	6.56	(3.78, 9.34)	0.000	-
model 1	4.51	(−0.61, 9.64)	0.080	-
model 2	3.62	(−1.08, 8.32)	0.119	0.048

The crude model included baseline depressive tendency alone. Model 1 additionally adjusted for the corresponding baseline outcome score. Model 2 additionally adjusted for age group and sex. Model 2 confidence intervals and p values were calculated using heteroskedasticity-robust standard errors. Age group was entered as an ordinal variable coded 1, 2, and 3 for ages 18–44, 45–64, and ≥65 years, respectively. Incremental *R*^2^ indicates the additional proportion of outcome variance explained by baseline depressive tendency in Model 2.

## Data Availability

The data presented in this study are available on request from the corresponding author due to privacy and ethical restrictions.

## Supplementary Materials

The following supporting information can be downloaded at https://www.mdpi.com/article/10.3390/healthcare14162577/s1, Table S1. Variance inflation factors for the fully adjusted regression models of post-counseling clinical outcomes; Table S2. PHQ-9-specific sensitivity analyses using alternative representations of baseline depression.

## Abbreviations

The following abbreviations are used in this manuscript:

AVE	Average variance extracted
CR	Composite reliability
DSM-IV	Diagnostic and Statistical Manual of Mental Disorders-IV
mBMI	The indicator of mental health BMI on well-being
MG	Myasthenia gravis
MG-ADL	Myasthenia Gravis Activities of Daily Living Scale
MG-QOL15	Myasthenia Gravis Quality of Life 15-item
*N*	Number of participants
PHQ-9	Patient Health Questionnaire-9
QMG	Quantitative Myasthenia Gravis
