# Supplementary material for: Association of Baseline Depressive Tendency with Post-Counseling Clinical Status in Patients with Thymoma-Associated Myasthenia Gravis
Source: Healthcare (Basel). 2026 Aug 17;14(16):2577. doi: 10.3390/healthcare14162577 (PMC13512784; doi:10.3390/healthcare14162577)
Supplement: Supplementary file 1 [file healthcare-14-02577-s001.zip › healthcare-4398913-supplementary.pdf]

## Supplementary materials

**Table S1.** Variance inflation factors for the fully adjusted regression models of post-counseling clinical outcomes

| Outcome model | Baseline depressive tendency | Corresponding baseline outcome score | Age group | Sex | Mean VIF |
|---|---|---|---|---|---|
| MG-ADL | 1.60 | 1.53 | 1.14 | 1.26 | 1.38 |
| MG-QoL15 | 2.29 | 2.45 | 1.25 | 1.12 | 1.78 |
| mBMI | 1.43 | 1.30 | 1.16 | 1.12 | 1.25 |
| PHQ-9 | 4.00 | 3.68 | 1.18 | 1.20 | 2.52 |

* VIF, variance inflation factor; MG-ADL, Myasthenia Gravis Activities of Daily Living scale; MG-QoL15, Myasthenia Gravis Quality of Life 15-item scale; mBMI, mental BMI; PHQ-9, Patient Health Questionnaire-9. Each fully adjusted Model 2 included baseline depressive tendency, the corresponding continuous baseline outcome score, age group, and sex. Age group was entered as an ordinal variable coded 1, 2, and 3 for ages 18–44, 45–64, and ≥65 years, respectively. Mean VIF was calculated across the four explanatory variables in each model. In the PHQ-9 model, baseline depressive tendency was derived directly from continuous baseline PHQ-9 using a cutoff score of ≥10.

**Table S2.** PHQ-9-specific sensitivity analyses using alternative representations of baseline depression

| Model and explanatory variable | β | Robust 95% CI | p value | Model $R^2$ | VIF |
|---|---|---|---|---|---|
| **Sensitivity Model A** | | | | **0.739** | |
| Baseline depressive tendency | 6.20 | 3.36 to 9.05 | <0.001 | | 1.10 |
| Age group | 1.97 | −0.77 to 4.70 | 0.145 | | 1.13 |
| Sex | −0.99 | −3.27 to 1.29 | 0.364 | | 1.12 |
| **Sensitivity Model B** | | | | **0.725** | |
| Continuous baseline PHQ-9 | 0.74 | 0.48 to 1.01 | <0.001 | | 1.01 |
| Age group | 2.71 | −0.36 to 5.79 | 0.078 | | 1.07 |
| Sex | 0.26 | −1.77 to 2.29 | 0.790 | | 1.06 |

* β represents the adjusted regression coefficient for post-counseling PHQ-9. Sensitivity Model A included baseline depressive tendency, age group, and sex and excluded continuous baseline PHQ-9. Sensitivity Model B included continuous baseline PHQ-9, age group, and sex and excluded the binary depressive-tendency indicator. Baseline depressive tendency was defined as a baseline PHQ-9 score ≥10. Age group was entered as an ordinal variable coded 1, 2, and 3 for ages 18–44, 45–64, and ≥65 years, respectively. Confidence intervals and p values were calculated using heteroskedasticity-robust standard errors. The sensitivity analyses were conducted to assess covariate-specification dependence and were not intended to replace the primary fully adjusted Model 2. CI, confidence interval; VIF, variance inflation factor; PHQ-9, Patient Health Questionnaire-9.
